# Self-reported activities of daily living, health and quality of life among older adults in South Africa and Uganda: a cross sectional study

**DOI:** 10.1186/s12877-020-01809-z

**Published:** 2020-10-14

**Authors:** Sanni Yaya, Dina Idriss-Wheeler, N’doh Ashken Sanogo, Maude Vezina, Ghose Bishwajit

**Affiliations:** 1 School of International Development and Global Studies, Faculty of Social Sciences, University of Ottawa, 120 University Private, Ottawa, ON K1N 6N5 Canada; 2 The George Institute for Global Health, Imperial College London, London, United Kingdom; 3 Interdisciplinary School of Health Sciences, Faculty of Health Sciences, University of Ottawa, Ottawa, Canada

**Keywords:** Activities of daily living, Health, Quality of life, Elderly health

## Abstract

**Background:**

Difficulties in performing the activities of daily living (ADL) are common among middle-aged and older adults. Inability to perform the basic tasks as well as increased healthcare expenditure and dependence on care can have debilitating effects on health and quality of life. The objective of this study was to examine the relationship between self-reported difficulty in activities of daily living (ADL), health and quality of life among community-dwelling, older population in South Africa and Uganda.

**Methods:**

We analyzed cross-sectional data on 1495 men and women from South Africa (*n* = 514) and Uganda (*n* = 981) which were extracted from the SAGE Well-Being of Older People Study (WOPS 2011–13). Outcome variables were self-reported health and quality of life (QoL). Difficulty in ADL was assessed by self-reported answers on 12 different questions covering various physical and cognitive aspects. The association between self-reported health and quality of life with ADL difficulties was calculated by using multivariable logistic regression models.

**Results:**

Overall percentage of good health and good quality of life was 40.4% and 20%, respectively. The percentage of respondents who had 1–3, 3–6, > 6 ADL difficulties were 42.4%7, 30.97% and 14.85%, respectively. In South Africa, having > 6 ADL difficulties was associated with lower odds of good health among men [Odds ratio = 0.331, 95%CI = 0.245,0.448] and quality of life among men [Odds ratio = 0.609, 95%CI = 0.424,0.874] and women [Odds ratio = 0.129, 95%CI = 0.0697,0.240]. In Uganda, having > 6 ADL difficulties was associated lower odds of good health [Odds ratio = 0.364, 95%CI = 0.159,0.835] and quality of life [Odds ratio = 0.584, 95%CI = 0.357,0.954].

**Conclusion:**

This study concludes that difficulty in ADL has a significant negative association with health and quality of life among community-dwelling older population (> 50 years) in South Africa and Uganda. The sex differences support previous findings on differential health outcomes among men and women, and underline the importance of designing sex-specific health intervention programs.

## Background

An aging population is fast becoming a public health concern in low-income settings and increasing demands on already constrained healthcare resources [1–4]. In the context of Sub-Saharan Africa, which has the youngest population worldwide, little work has been done to examine the health, quality of life and well-being of the older population. While geriatric care is well-developed in high-income settings, this particular area of public health has received less attention than it merits, especially in Africa [3, 5, 6]. The apparent oversight regarding elderly healthcare can be attributed to inadequate research and financial capacities as well as the immediate and significant burden of malnutrition and communicable diseases which require urgent intervention [7–9].

In recent decades, African countries, on average, have experienced substantial improvements in terms of declining maternal and child mortality, higher rates of child survival, and subsequent improvements in life expectancy [10–13]. These slow but steady alterations in the epidemiological profile have coincided with a sharp rise in the prevalence of non-communicable chronic diseases (NCDs) [14–17]. Another noticeable change that characterizes the African demographic transition in recent decades is the concomitant rise in the number of elderly in the populations [3, 18]. While the proportion of older population relative to younger population remained the same, or even decreased because of high fertility rates in the region, the actual number of the elderly in the population has been growing yet issues surrounding their healthcare remain an underreported area in research.

Aging is a natural process, and the provision of care to the elderly, irrespective of their position in the labor market, is a marker of good quality of living and welfare policies, as well as national progress at large. Unfortunately, inadequate healthcare infrastructure, insufficient financial capacity, lack of research and little political attention hinder the progress of geriatric medicine in low-resource countries [19–21]. Changing demographic and disease profiles require subsequent restructuring in the care delivery model to meet the changing health needs of the population. Given the increased susceptibility to NCDs among the elderly, and the constant expansion of NCDs in the populations, healthcare systems need to reorient the model of care from predominantly curative to more promotive and preventive services [22–24]. In addition to preventive care, the psychosocial needs of older populations have also been a commonly overlooked aspect of healthcare services in Africa. Promoting elderly health and enhancing quality of life outcomes is becoming challenging; this is due to the complex interplay of an increasingly expanding set of forces within the environmental, social, economic and political contexts [25–29].

An effective way of dealing with the complex set of risk factors of health and diseases among the elderly has been set out in the biopsychosocial model of care [30–32]. This model acknowledges the diverse health, social and psychological needs that are necessary to ensure optimum health status and quality of life not only among elderly, but among younger populations as well. Not only is the provision of quality medical care for the elderly necessary, but also the establishment of a healthy living environment and adequate social support [33]. This is particularly important because of increased susceptibility of old aged individuals to developing functional disabilities. People living with disabilities share an increased risk of malnutrition, loneliness, psychological morbidity and frequent hospitalisation [34–36]. From this perspective, studies on ADL disability and its association with health status can provide important insights to help caregivers and clinicians work with older patients more effectively, especially for individuals living with difficulties in performing instrumental activities of daily living [37]. Currently, the literature on health status among older population living with ADL difficulties is extremely scarce for countries in Africa. We aim to address this research gap by using data on elderly health from South Africa and Uganda. The data were collected by the World Health Organization (WHO) under the Well-Being of Older People Study (WOPS) project conducted between 2009 and 2013. The main objective was to examine whether self-reported health and quality of life differ significantly among older men and women (> 50 Years of age) living with difficulties in core ADLs compared with individuals free from ADL difficulties. The insights generated can help geriatric practitioners and researchers in these two countries and others with similar demographic and socioeconomic profiles.

## Methods

### Data source

Data used in this survey were obtained from the WHO SAGE Well-Being of Older People Study (WOPS). These were sub-population surveys carried out between 2010 (Wave 1) and 2013 (Wave 2) in Uganda and South Africa, in partnership with the Medical Research Council/Uganda Virus Research Unit, Uganda Research Unit on AIDS, Uganda, and the Africa Centre Demographic Information System (ACDIS) and population-based HIV survey, South Africa [38]. The objectives of these surveys were to provide data on the various health, demographic and social indicators relevant to the health and functional status among older people either infected with HIV themselves, or affected by HIV/AIDS in their families. Details of sampling procedures and study protocols were published as WHO reports [39].

### Measures

The outcome measures of this study were: 1) self-reported health (SRH), and 2) self-reported quality of life (QoL). These two were assessed by single-item questions: 1) SRH: “How satisfied are you with your health?” and 2) QoL: How would you rate your overall quality of life?” The answers ranged from: Very Good, Good, Moderate, Bad and Very Bad. Participants were classified as having good SRH if they responded “Very Good or Good”, and not good SRH if they responded otherwise. Quality of life was categorized in the same manner.

Validity of these single-item instruments for assessing health [40, 41] and quality of life [42, 43] were established in various settings, and used across many research domains because of ease and cost-effectiveness in measurement, as well as strong predictability for morbidity and mortality [44].

Difficulty in ADL was assessed by the following 12 questions surrounding physical and cognitive aspects of disability - In the last 30 days/month, how much difficulty did you have with: 1) moving around, 2) concentrating and remembering things, 3) learning a new task, 4) standing for long time, 5) bathing/washing, 6) getting dressed, 7) carrying things, 8) eating, 9) getting up from lying, 10) using toilet, 11) using public transportation, 12) going out. The possible answers were: 1. None, 2. Mild, 3. Moderate, 4. Severe, and 5. Extreme. Those who reported no difficulty were classified as “No functional difficulties”, and as “Has functional difficulties” if reported otherwise. The number of ADL difficulties were categorized as: None,1–3, 4–6, and > 6.

Variables such as health and quality of life status are multifactorial and are thought of as a complex outcome of a wide range of demographic, social and environmental circumstances. In light of this understanding, we included several other variables as potentially confounding factors: Age (50–59, 60–69, 70–79, 79+ years); Sex (female, male); Current marital status (married, not married); Religion (Christian, Islam/Other); Ever used tobacco (Yes, no); Ever alcohol consumption (Yes, no); Living condition (Very Satisfied, Satisfied, Neither satisfied nor dissatisfied, Dissatisfied, Very dissatisfied); Sleep difficulty (None, Mild/Moderate, Severe/Extreme); Number of chronic multimorbidity (0, 1, > 1). Multimorbidity was defined as having more than one chronic condition [45] assessed by self-reported diagnosis results: Arthritis, Asthma, Chronic Lung Disease, Diabetes, Cataract, Heart Disease, Stroke, Hypertension. Older age was defined as per the WHO guideline [46].

### Data analysis

Data analyses were carried out using STATA version 14. Firstly, we ran a set of descriptive statistics to present sample characteristics and the prevalence of depression and fatigue. Given the sociocultural heterogeneity of the sample populations, we reported the data for South Africa and Uganda separately throughout the analysis. Further, we also report the results separately by sex as recommended for population studies. Next, we ran three sets of multivariate models for each country: one for the pooled sample, one for the male sample and one for the female sample. The results of the regression analysis were presented as odds ratios using 95% confidence intervals (CIs). A two-tailed *P*-value of < 0.05 was set as level of significance for all calculations.

### Ethics statement

The WOPS survey was approved by the implementing bodies in the respective countries. The datasets were made available in the public data repository of the WHO in anonymized form, hence no further approval was necessary for this study.

## Results

### Descriptive statistics

Sample characteristics are presented in Table 1. A greater percentage of the participants were aged 50–59 years, female, currently unmarried, and were followers of Christianity. About three-fifth (58.7%) reported their living condition as satisfactory. Lifetime prevalence of tobacco and alcohol use was 25.7% and 57.8%, respectively. Just under three quarters (71.6%) of the participants had no chronic conditions, whereas 7.6% were suffering from more than two chronic conditions. Mild/Moderate and Severe/Extreme sleep difficulties were reported by 32.2% and 23.1% participants, respectively. Two fifth of the participants rated their health as good, and one-fifth reported having good quality of life.

**Table 1:** Participant characteristics. SAGE WOPS 2010–13

	Description	***N*** = 1495	Percentage
**Country**			
South Africa	Country of survey	514	34.4
Uganda		981	65.6
**Age groups**			
50–59	Current age of the participants	860	57.5
60–69		301	20.1
70–79		232	15.5
79+		102	6.8
**Sex**			
Male	Sexual orientation	722	48.3
Female		773	51.7
**Marital status**			
Not Married	Current living arrangement	312	20.9
Married/Cohabitating		1183	79.1
**Religion**			
Catholic	Religious affiliation	1086	72.6
Islam/Other		409	27.4
**Living condition**			
Satisfactory	Self-reported situation of living environment	870	58.7
Neural		426	28.5
Not Satisfactory		199	12.8
**Tobacco**			
Yes	History of tobacco use	387	25.7
No		1108	74.3
**Alcohol**			
Yes	History of alcohol use	865	57.8
No		629	42.2
**Multimorbidity**			
0	Total number of diagnosed NCDs	1070	71.6
1		311	20.8
> 1		114	7.6
**Sleep difficulty**			
None	Self-reported difficulty in falling asleep	669	44.7
Mild/Moderate		481	32.2
Severe/Extreme		345	23.1
**SRH**			
Good	Self-rated health status today	604	40.4
Moderate		624	41.7
Bad		267	17.9
**QoL**			
Good	Self-reported quality of life	299	20.0
Moderate		685	45.8
Bad		497	33.2
**No. of ADL difficulties**			
None	Number of self-reported difficulties	175	11.71
1–3		635	42.47
4–6		463	30.97
> 6		222	14.85

**Fig. 1:**
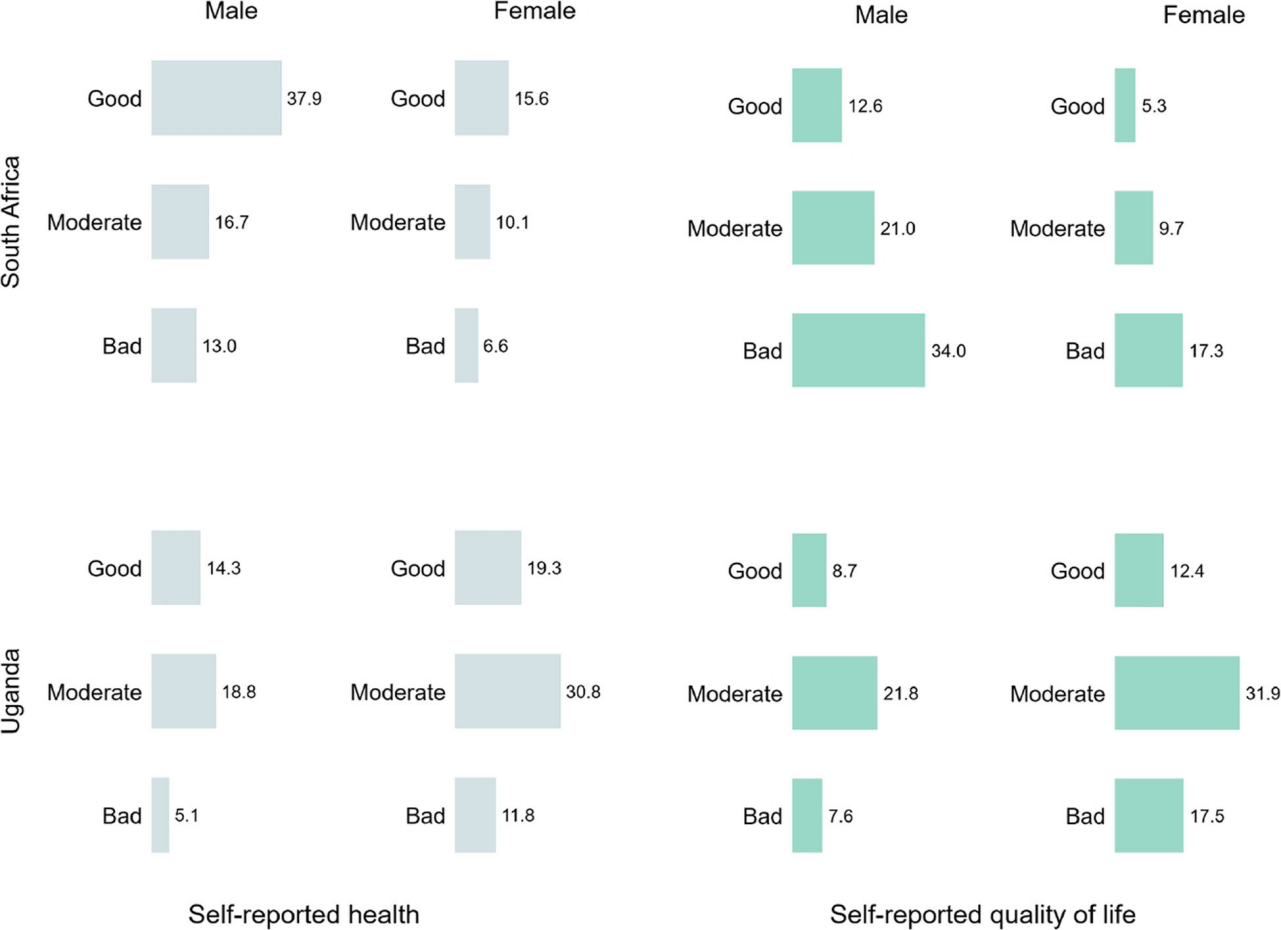
Country-wise percentage of self-reported health and quality of life by sex

Figure 1 shows the percent distribution of the categories of health and quality of life for men and women in South Africa and Uganda. In South Africa, the percentage reporting good health (37.9% vs. 15.6%) and quality of life (12.6% vs. 5.3%) was higher among men compared to women. Whereas in Uganda, women were more likely to report good health (19.3% vs. 14.3%) and quality of life (12.4% vs. 8.7%) compared to the men.

**Fig. 2:**
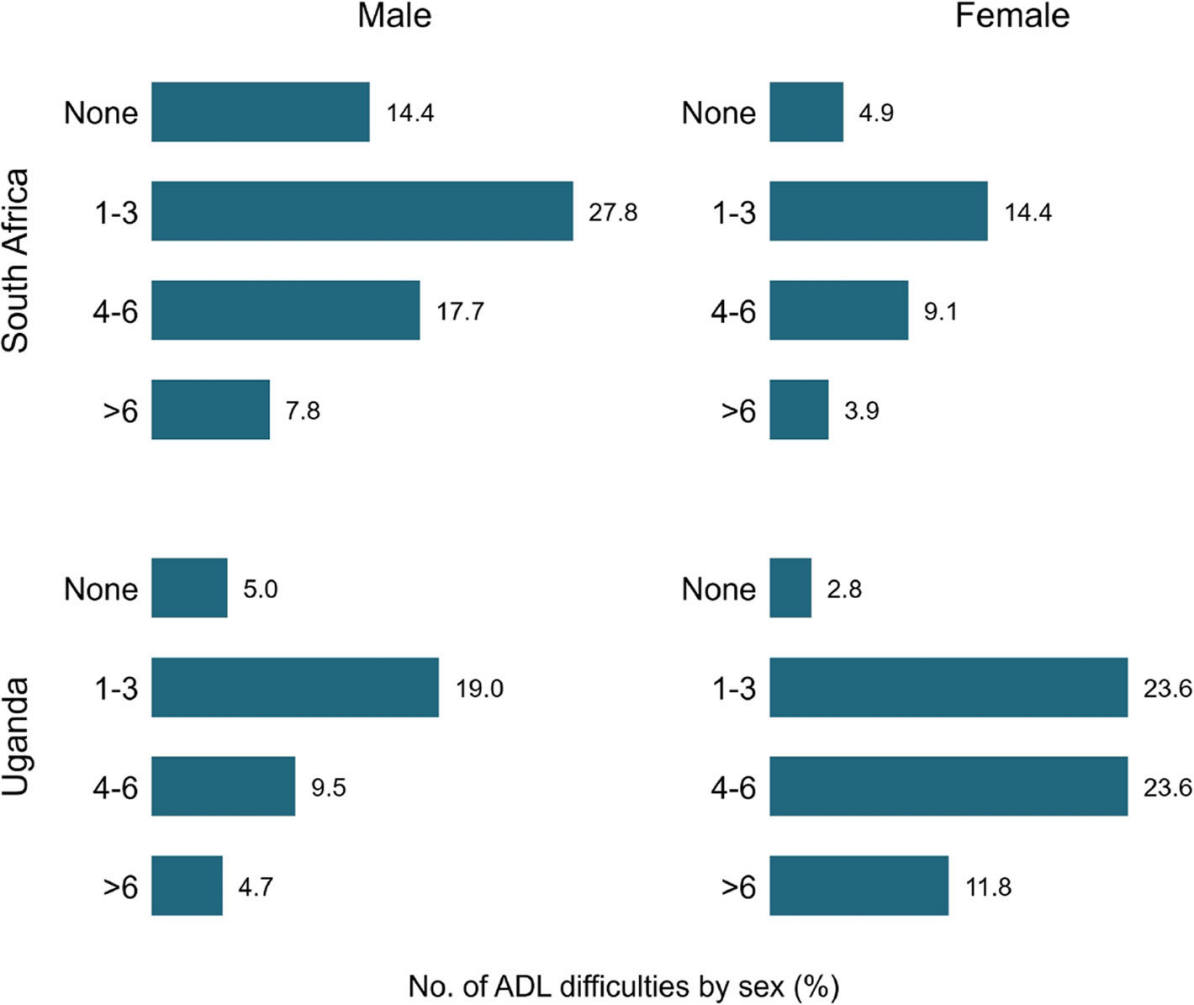
Sex-differences in reporting ADL difficulties

Figure 2 also shows the percent distribution of ADL difficulties among men and women in South Africa and Uganda. In south Africa, 14.4% of the men reported having no difficulties compared with 4.9% of women. The percentage of reporting 1–3, 4–6 and > 6 ADL difficulties were higher among men in South Africa, while in Uganda the respective percentages were higher among women.

Table 2 summarizes the results of the association between self-reported health and quality of life with ADL difficulties in South Africa.

**Table 2:** Association between ADL with self-reported health (SRH) and quality of life (QoL) in South Africa

	SRH			QoL		
	Overall	Men	Women	Overall	Men	Women
**Age (**50–59**)**						
60–69	0.913	0.964	0.873	0.765	0.710	0.883
	[0.639,1.306]	[0.630,1.475]	[0.578,1.320]	[0.414,1.413]	[0.172,2.941]	[0.588,1.325]
70–79	0.598^*^	0.616	0.440^**^	0.567^**^	0.726	0.454^***^
	[0.382,0.937]	[0.371,1.022]	[0.248,0.780]	[0.402,0.799]	[0.423,1.247]	[0.287,0.719]
80+	0.850	0.542	0.475^**^	0.709	0.449	0.453^**^
	[0.379,1.908]	[0.280,1.047]	[0.295,0.764]	[0.358,1.405]	[0.188,1.076]	[0.253,0.813]
**Sex (Male)**						
Female	1.225			1.042		
	[0.821,1.828]			[0.715,1.519]		
**Currently married (No)**						
Yes	2.620^***^	1.927^**^	1.052	1.470	1.545	1.061
	[1.647,4.168]	[1.274,2.915]	[0.721,1.534]	[0.837,2.582]	[0.761,3.134]	[0.565,1.992]
**Religion (Christian)**						
Islam/Other	1.334	0.564^*^	1.031	1.165	0.538	1.026
	[0.827,2.151]	[0.325,0.978]	[0.581,1.829]	[0.534,2.541]	[0.188,1.540]	[0.575,1.832]
**Smokes (No)**						
Yes	1.078	0.883	0.942	0.901	0.882	0.688
	[0.747,1.555]	[0.588,1.325]	[0.609,1.459]	[0.630,1.287]	[0.553,1.407]	[0.0363,13.01]
**Alcohol (No)**						
Yes	0.926	1.063	1.135	0.444	0.548	0.742
	[0.612,1.402]	[0.682,1.658]	[0.697,1.847]	[0.0148,13.31]	[0.204,1.472]	[0.370,1.489]
**Living condition (Satisfactory)**						
Neutral	1.006	0.294^***^	0.254^***^	1.519	0.765	0.930
	[0.700,1.445]	[0.186,0.464]	[0.153,0.422]	[0.0772,29.91]	[0.414,1.413]	[0.0103,83.84]
Not satisfactory	1.441	0.818	0.492^*^	1.075	0.596	0.455^*^
	[0.898,2.313]	[0.490,1.365]	[0.271,0.894]	[0.711,1.626]	[0.310,1.145]	[0.214,0.967]
**Sleep problem (None)**						
Mild/Moderate	0.571^**^	0.864	0.419^***^	0.127^***^	0.191	0.0993^***^
	[0.404,0.806]	[0.572,1.303]	[0.274,0.642]	[0.0486,0.330]	[0.0383,1.504]	[0.0291,0.339]
Severe/Extreme	0.490^**^	1.114	0.488^*^	0.591^*^	0.900	0.378^**^
	[0.304,0.790]	[0.601,2.065]	[0.279,0.853]	[0.378,0.924]	[0.489,1.656]	[0.192,0.745]
**Multimorbidity (0)**	1	1	1			
1	0.723	1.087	0.756	0.734	0.712	0.742
	[0.511,1.023]	[0.741,1.595]	[0.480,1.191]	[0.519,1.038]	[0.406,1.250]	[0.470,1.171]
> 1	0.866	1.435	0.889	0.884	0.961	0.869
	[0.509,1.475]	[0.846,2.435]	[0.566,1.397]	[0.519,1.504]	[0.378,2.445]	[0.450,1.678]
**ADL**						
1–3	0.273^***^	0.774	0.129^***^	0.263^**^	0.490	0.972
	[0.147,0.506]	[0.553,1.084]	[0.0697,0.240]	[0.0981,0.705]	[0.203,1.181]	[0.415,2.277]
4–6	0.586^**^	0.121^***^	0.468	0.856	0.488^***^	0.251^***^
	[0.420,0.817]	[0.0532,0.276]	[0.0233,9.398]	[0.463,1.581]	[0.338,0.705]	[0.169,0.374]
> 6	0.477^***^	0.331^***^	0.820	0.404	0.609^**^	0.129^***^
	[0.313,0.727]	[0.245,0.448]	[0.455,1.476]	[0.156,1.050]	[0.424,0.874]	[0.0697,0.240]

Exponentiated coefficients; 95% confidence intervals in brackets

^*^ *p* < 0.05, ^**^ *p* < 0.01, ^***^ *p* < 0.001

Compared with participants in the highest age category, those in the lower age groups had relatively higher odds of reporting good health and quality of life, especially among women. In South Africa, women aged 80+ years had lower odds of reporting good health [0.475,0.295,0.764] and quality of life [0.453,0.253,0.813]. Women who reported living condition as not satisfactory also had lower odds of reporting good health [0.492, 0.271,0.894] and quality of life [0.455, 0.214,0.967]. Mild/moderate and severe/extreme sleep difficulty were also associated with lower odds of reporting good health and quality of life among women. Compared with those with no ADL difficulties, those who had any number of difficulties had significantly lower odds of reporting good health and quality of life. Having > 6 ADL difficulties were associated lower odds of good health among men [Odds ratio = 0.331, 95%CI = 0.245,0.448] and quality of life among men [Odds ratio = 0.609, 95%CI = 0.424,0.874] and women [Odds ratio = 0.129, 95%CI = 0.0697,0.240].

Table 3 summarizes the results of the association between self-reported health and quality of life with ADL difficulties in Uganda. The association between age and health and quality of life was not significant among women. Men aged 70–79 [Odds ratio = 0.273, 95%CI = .147,0.612] and 80+ [Odds ratio = 0.141, 95%CI = 0.0254,0.783] years had lower odds of reporting good quality of life. Women who reported severe/extreme sleep difficulty had lower odds of reporting good health [Odds ratio = 0.138, 95%CI = 0.0215,0.883]. Having ADL difficulties showed a significantly negative effect on good health and quality of life. Having > 6 ADL difficulties were associated lower odds of good health [Odds ratio = 0.364, 95%CI = 0.159,0.835] and quality of life [Odds ratio = 0.584, 95%CI = 0.357,0.954].

**Table 3:** Association between ADL with self-reported health (SRH) and quality of life (QoL) in Uganda

	SRH			QoL		
	Overall	Men	Women	Overall	Men	Women
**Age (50–59)**						
60–69	0.862	0.930	1.023	0.921	1.412	0.753
	[0.487,1.523]	[0.402,2.150]	[0.411,2.546]	[0.553,1.536]	[0.580,3.437]	[0.379,1.497]
70–79	0.604^*^	0.471	0.692	0.508^*^	0.273^***^	1.047
	[0.370,0.985]	[0.208,1.064]	[0.361,1.326]	[0.262,0.986]	[0.147,0.612]	[0.407,2.695]
80+	0.878	1.903	0.568	0.388	0.141^*^	0.819
	[0.412,1.871]	[0.482,7.518]	[0.208,1.552]	[0.136,1.105]	[0.0254,0.783]	[0.213,3.159]
**Sex (Male)**						
Female	1.354			1.913		
	[0.663,2.763]			[0.842,4.347]		
**Currently married (No)**						
Yes	0.635	0.793	0.465	0.703	0.697	0.619
	[0.324,1.242]	[0.242,2.598]	[0.193,1.124]	[0.415,1.191]	[0.281,1.729]	[0.310,1.237]
**Religion (Christian)**						
Islam/Other	0.849	0.560	0.448	0.659	0.751	0.305
	[0.254,2.845]	[0.176,1.776]	[0.0865,2.324]	[0.354,1.226]	[0.354,1.591]	[0.0774,1.205]
**Smokes (No)**						
Yes	0.841	1.087	0.803	0.570	0.485	1.500
	[0.513,1.377]	[0.461,2.565]	[0.419,1.538]	[0.299,1.086]	[0.148,1.585]	[0.663,3.391]
**Alcohol (No)**						
Yes	0.827	1.558	0.571	1.338	0.367	1.238
	[0.469,1.458]	[0.867,2.799]	[0.0202,16.17]	[0.659,2.717]	[0.0135,9.941]	[0.601,2.549]
**Living condition (No satisfactory)**						
Neutral	0.502	0.498	1.114	0.900	0.735	1.142
	[0.0187,13.46]	[0.175,1.419]	[0.0580,21.39]	[0.534,1.517]	[0.339,1.593]	[0.513,2.544]
Satisfactory	0.822	0.662	1.380	1.292	1.821	1.215
	[0.359,1.883]	[0.258,1.703]	[0.202,9.437]	[0.635,2.630]	[0.579,5.727]	[0.434,3.402]
**Sleep problem (None)**						
Mild/Moderate	0.253	0.943	0.655	0.750	0.445	1.020
	[0.0109,5.862]	[0.653,1.360]	[0.00717,59.82]	[0.465,1.208]	[0.195,1.017]	[0.552,1.882]
Severe/Extreme	0.183^**^	0.222	0.138^*^	0.573	0.736	0.428
	[0.0605,0.553]	[0.0480,1.025]	[0.0215,0.883]	[0.205,1.606]	[0.199,2.715]	[0.0700,2.623]
**Multimorbidity (0)**						
1	1.880^*^	1.864	3.391^*^	1.004	0.967	1.128
	[1.038,3.404]	[0.0865,40.17]	[1.038,11.08]	[0.699,1.441]	[0.538,1.739]	[0.704,1.808]
> 1	0.623	1.079	1.446	1.317	1.456	2.615
	[0.272,1.428]	[0.280,4.159]	[0.902,2.320]	[0.647,2.680]	[0.761,2.786]	[0.951,7.188]
**ADL**						
1–3	0.490^**^	0.520	0.425^*^	0.314^*^	0.268^***^	0.399^*^
	[0.298,0.805]	[0.236,1.145]	[0.216,0.833]	[0.110,0.897]	[0.145,0.496]	[0.192,0.830]
4–6	0.378^**^	0.166^*^	0.462	0.131^**^	0.0764^***^	0.145^***^
	[0.184,0.775]	[0.0310,0.886]	[0.200,1.066]	[0.0378,0.454]	[0.0390,0.149]	[0.0597,0.353]
> 6	0.364^*^	0.645	0.619^*^	0.584^*^	0.627	0.716
	[0.159,0.835]	[0.361,1.153]	[0.393,0.975]	[0.357,0.954]	[0.344,1.142]	[0.427,1.202]

Exponentiated coefficients; 95% confidence intervals in brackets

^*^ *p* < 0.05, ^**^ *p* < 0.01, ^***^ *p* < 0.001

## Discussion

Sub-Saharan African countries, such as South Africa [47, 48] and Uganda [49, 50] are recognizing the importance and challenge of health issues associated with poor health and disability among the aging population. Difficulties in performing basic activities such as cooking, washing, and walking are more common among middle-aged and older adults, affecting their general health and quality of life, and consequently increasing their dependence on care. In this study, we assessed the relationship between self-reported difficulty in Activities of Daily Living (ADL), health and quality of life among community-dwelling population aged 50 years and above in South Africa and Uganda. We found that only two-fifth of the participants reported having good health and one-fifth reported good quality of life. The analysis revealed disparities of varying degrees in the prevalence of good health and quality of life between men and women in the two countries. In general, the percentage reporting good health and quality of life was higher among South African men, with the opposite findings in Uganda where more women reported good health and quality of life than men. Sex differences in health and quality of life were observed in previous studies as well [51, 52]. Although the main causes leading to this disparity remain unclear, the difference is generally thought to be rooted in the way men and women perceive health and illness and communicate their health issues [53, 54]. In light of the existing literature, individual’s overall wellbeing is reliant on meeting the physical and psychological needs which itself is greatly influenced by sociocultural factors [52]. In the context of Africa, as well as in many other underdeveloped countries and regions, women’s lower subjective health and quality of life can be explained by their lower socioeconomic standing and the state of gender inequality [52].

Another possible reason for sex-differences can be exposure to risk factors that underlie health and illness conditions. In most African societies, men and women are assigned gender-specific familial and social roles which can determine their degree of vulnerability to certain sources or types of disability. For instance, men are more likely to engage in physically demanding jobs than women, which consequently increase their risk of morbidity and mortality. A systematic review and meta-analysis reported that men are more prone to early death compared to women, which can be explained by their engagement in physically strenuous professions [55]. Interestingly, in this study, we found that men had a higher percentage of reporting no difficulties in both of the countries, but regardless the percentage of reporting 1–3, 4–6 and > 6 ADL difficulties were also higher among South African men. In contrast, the percentage of having a larger number of ADL difficulties was higher among women in Uganda, implying the presence of unobserved factors that are responsible for this difference. The association between sex and ADL difficulties was lost after adjusting for other covariates. Nonetheless, in the sex-stratified analysis we noticed important differences in the association between ADL difficulties and self-reported health and quality of life. For instance, in South Africa, the larger number of ADL difficulties decreased the odds of health and quality of life for both men and women. However, the association with health was statistically significant only among men. On the other hand, having the higher number of ADL difficulties (> 6) was associated only with health among men but not women, while no association was found with quality of life in Uganda.

Findings reveal a negative impact of ADL difficulties on health and quality of life, with varying trends among men and women. From this perspective, it is recommended that health intervention programs carry out sex-specific investigations on general health outcomes. The advantage of this approach is a more nuanced intervention technique which can address the contextual determinants of health needs and risk factors among men and women. For example, women’s reproductive health issues are generally considered to be of larger concern than men’s since it is associated with the health outcomes of the offspring. Women also account for the bulk of care-giving responsibilities for children and other family members which can expose them to health issues which differ from men. As this was a secondary study, we are not able to adjust the analysis for these factors which could allow for a better understanding of the underlying meaning of the findings. Investigation using qualitative studies may be more suitable for exploring the gender differences and subjective health outcomes. The findings of the present study should be interpreted with caution since the data are cross-sectional. More updated evidence from primary research will be necessary on elderly population health in the areas of chronic diseases and disabilities to ensure better management of health among people living with difficulties in ADL.

An important contribution of this study is the use of subjective measures of health and quality of life which are more common among researchers in high-income countries. Empirical evidence on the use of these measures to assess general health status can serve as a new and effective way to perceive, investigate, and treat health in these countries. This study advances the literature in in the use of subjective measures of health and quality of life to explore their association with difficulties in ADL; another understudied subject in African population health.

There are limitations of the subjective constructs in population health studies. Being a social construct, some people may report their living standard as good, even if they feel otherwise, in order to provide a more socially acceptable answer. However, reporting bias is common in all health related behaviour such as smoking and alcohol drinking. The findings should be interpreted with caution because the data are self-reported which increases the chance of reporting bias. For instance, older people are more likely to have cognitive difficulties which could result in incorrect estimation of their ADL level. More studies are required to assess the suitability of the subjective constructs of health and quality of life in African populations. This was a cross-sectional study and therefore no causal relationship between the outcome and explanatory factors can be stated. The direction of the relationship is rather intuitive as difficulty in maintaining ADL is most likely the cause of poor health, but not vice versa. It is also important to note that the choice of variables for the analysis was limited by the secondary nature of the data. Several important factors such as cognitive functions were not adjusted for in the analysis. The variables were chosen in light of their demonstrated and conceptual association with the outcomes.

## Conclusion

A remarkably low percentage of 50+ years men and women in Uganda and South Africa reported having good health and quality of life. Important sex and country level differences were observed in the percentages which require further qualitative investigation. Having ADL difficulties was found to be a significant predictor of poor health and quality of life in both countries. More nuanced understanding of the associations between ADL difficulties and subjective health and quality of life may facilitate planning more effective health intervention programs for the growing number of older populations in Uganda and South Africa. Future studies should focus on self-management techniques among people living with ADL disabilities with an aim to promote their health and quality of life.

## Data Availability

Data for this study were sourced from the SAGE Well-being of Older People Study (WOPS) available at: http://apps.who.int/healthinfo/systems/surveydata/index.php/catalog/wops

## Abbreviations

ADL: Activities of Daily Living
NCDs: Non-communicable chronic diseases
SHR: Self-reported health
QoL: Self-reported quality of life
